# Treatment patterns of patients diagnosed with major depressive disorder and suicidal ideation or attempt: a U.S. population-based study utilizing real-world data

**DOI:** 10.1186/s12888-021-03616-1

**Published:** 2021-12-06

**Authors:** David M. Kern, M. Soledad Cepeda, Frank Wiegand

**Affiliations:** 1 Janssen Research & Development, LLC, 1125 Trenton Harbourton Rd, Titusville, NJ 08560 USA; 2 Janssen Global Services LLC, Titusville, NJ 08560 USA

**Keywords:** Suicidal ideation, Suicide attempt, Major depressive disorder, Treatment patterns, Administrative claims

## Abstract

**Background:**

There is a knowledge gap regarding the treatment patterns of patients with major depressive disorder (MDD) who experience suicidal ideation or a suicide attempt (SI/SA).

**Methods:**

Patients with SI/SA were identified from a large US-based claims database covering 84 million lives, during 1/1/2014–3/31/2020. Patients with MDD were indexed at their first diagnosis for SI/SA and followed up to 365 days. Treatment patterns were captured at the class level and included procedures of electroconvulsive therapy and transcranial magnetic stimulation, and pharmacotherapy including selective serotonin reuptake inhibitors (SSRIs), serotonin and norepinephrine reuptake inhibitors, tricyclic antidepressants, other antidepressants, anxiolytics, hypnotics/sedatives, antipsychotics, psychostimulants, and lithium.

**Results:**

There were 42,204 MDD + SI/SA patients identified. In the year prior to the index event > 40% of individuals received an SSRI and more than one-third received an anxiolytic. Within 1 year following, 84.4% received ≥1 of the treatments of interest. Of those, 70.2% went on to a subsequent class-based regimen, 46.3% received a third, and 28.1% received ≥4. More than three-quarters of patients received multiple treatment classes simultaneously. SSRIs were the most common treatments during follow-up (61.9%), followed by other antidepressants (51.3%), anxiolytics (50.8%) and anticonvulsants (43.6%).

**Conclusions:**

There was a large amount of variability and polypharmacy in the treatments received by MDD patients with SI/SA, and is much more complex than what has been previously observed in the general MDD population. Within one-year, many patients received four or more unique class-based regimens and most patients received treatments from multiple classes simultaneously, indicating the high unmet medical need and therapy refractoriness of this patient population.

**Supplementary Information:**

The online version contains supplementary material available at 10.1186/s12888-021-03616-1.

## Background

Major depressive disorder (MDD) affects more than 17 million adults and 3 million adolescents in the United States (US) [1]. Patients with MDD are at an increased risk of suicide, especially those with comorbid conditions such as anxiety and insomnia [2, 3]. Approximately one-quarter of patients diagnosed with MDD [4, 5], and 3–4% of the entire US adult population [6–8], experience suicidal thoughts (i.e., suicidal ideation), with more than 46,000 adults dying by suicide in the US each year [9]. The treatment of patients with depression with suicidal ideation or suicidal attempts is particularly challenging due to the urgency of the condition, the need to for immediate interventions to save lives and the limited treatment options. In addition, patients with suicidality suffer from poorer quality of life and social functioning than patients with MDD without suicidality [10–12]. Treating MDD patients contemplating suicide is a complicated problem that requires physicians to treat not just the depression, or to simply consider these patients as having a severe subtype of major depression, but instead to consider the suicidality itself as a distinct condition which warrants its own consideration [13]. In addition to finding an intervention that works quickly and effectively, it’s important for the clinician to understand why the patient wishes to commit suicide and create a trusting bond between the patient and the physician [14].

The recommended treatment for individuals diagnosed with MDD and having suicidal ideation is to treat the underlying depression [15, 16]. Treatment of the underlying MDD, includes not only antidepressants but also the use of other effective treatments for the depression and contributing comorbidities and symptoms, including lithium, atypical antipsychotics, antiepileptics, and other therapies [17–19].

Identifying patients at high risk of suicide, including those with suicidal ideation, is difficult in a primary care setting [20], and represents an opportunity to learn more about this population. Real-world data can serve as a very useful tool to understand this patient population. Research utilizing real-world data has been published detailing patient characteristics and treatment patterns of MDD patients in general [21], but similar data does not currently exist for the subset of those who have attempted suicide or have suicidal ideation.

This study leverages data from a large real-world US population to examine treatment patterns in the year following a suicide attempt or suicidal ideation of patients diagnosed with MDD. This work fills a current knowledge gap for a population that is understudied.

## Methods

### Data source

Data from this study came from the Optum© De-Identified Clinformatics® Data Mart Database. The Optum database is comprised of administrative insurance claims of more than 84 million Americans with private health insurance. The data includes those who are fully insured in commercial plans, those using administrative services only, and those insured with Medicare Advantage. Data were available from May 31, 2000 through March 31, 2020.

Data include outpatient pharmacy dispensing claims (using National Drug Codes) and inpatient and outpatient medical claims which contain diagnosis codes (ICD-9-CM or ICD-10-CM) and procedure codes (CPT, ICD-9-CM, and ICD-10-PCS). The use of the Optum claims database was reviewed by the New England Institutional Review Board (IRB) and was determined to be exempt from broad IRB approval, as this research project did not involve human subjects research.

### Patient identification

Patients diagnosed with suicidal ideation or a suicide attempt / self-harm (SI/SA) according to International Classification of Diseases, Clinical Modification codes, 9th and 10th revisions (ICD-9 and ICD-10) were identified. The algorithms used to identify suicide attempts are based on two validated algorithms which reported positive predictive values (PPV) ranging from 70 to 100% [22, 23], and corresponding ICD-10-CM codes were added to account for more recent data [24]. There exists one ICD-9-CM code (V62.84) and one ICD-10-CM code (R45.851) for suicidal ideation which were also added to the code list. The date of the first observation for SI/SA in the database was declared as the index date. This method for identifying SI/SA events has been used in previous research [25] with the exception that events in the current study were not limited to hospitalizations.

Patients met the diagnostic criteria for MDD if they had a diagnosis of depression on at least two distinct dates, or an inpatient hospitalization for depression, in the year prior to (and including) the index date. The MDD definition is based on the validated algorithm published by Solberg et al. [26] The full list of diagnoses used to identify SI/SA and MDD is found in Additional file 1. Patient data was obtained for the period of January 1, 2014 through March 31, 2020. Patients with a history of mania, psychosis, dementia, or autism were excluded and patients were required to have at least 365 days of continuous enrollment in the database prior to the index date. No minimum post-index observation was required and patients were followed up to a maximum of 365 days to capture treatment patterns.

### Treatment patterns

Treatment patterns were captured at the class level and included the pharmacotherapies of selective serotonin reuptake inhibitors (SSRIs), serotonin and norepinephrine reuptake inhibitors (SNRIs), tricyclic antidepressants, monoamine oxidase inhibitors (MAOIs), other antidepressants (including bupropion and trazodone, among others), anxiolytics, hypnotics/sedatives, antipsychotics, psychostimulants and lithium; and procedures of electroconvulsive therapy (ECT) and transcranial magnetic stimulation (TMS). The individual drugs, procedures, and their corresponding codes are found in Additional file 2.

Treatment sequences, referred to as “class-based regimens”, were captured from the index date through 365 days following. A single regimen included all medication classes of interest that were received by a patient concurrently – for example, if a patient simultaneously received an SSRI and an anxiolytic their class-based regimen was SSRI+anxiolytic. Any change to the class of medications received by a patient are reflected as a new treatment regimen. Only first use of a medication class was captured and not counted again in later lines of therapy – for example an individual filling an SSRI, switching to an antipsychotic, and then moving back to an SSRI would only be captured as switching from SSRI to an antipsychotic. Because the analysis is at the class-level, in-class switching and in-class combination therapy is not captured – for example, switching from one SSRI to another or adding an SSRI to an existing SSRI regimen was not observed. Combination therapy was captured when claims for the first occurrence of two different medication classes or procedures occurred simultaneously (within 14 days of each other). Thus, the treatment patterns reflect what new medication classes / procedures were being administered at the time a change in treatment class occurred.

### Patient characteristics and comorbidities

Patient characteristics captured include demographics (age, gender) on the index date, the Charlson comorbidity index, medication use and individual comorbid conditions for the year preceding the index date. Comorbid conditions required a single diagnosis and were defined using Systematized Nomenclature of Medicine - Clinical Terms (SNOMED CT) classification system [27, 28].

### Common data model

Data from all the database were mapped to standard concepts according to the Observational Medical Outcomes Partnership (OMOP) Common Data Model v5.0 [29] and the treatment sequence analysis was performed within the Observational Health Data Sciences and Informatics (OHDSI) framework.

## Results

There were 42,204 MDD + SI/SA patients identified from the database. Individuals were 45.4 years old on average and 57.2% were female. The mean (SD) Charlson Comorbidity Index score was 2.1 (3.1). More than half of patients were followed for the full 365-day post-index period, and the mean follow-up was 265 days (Table 1). Anxiety disorder was diagnosed in more than 40% of patients during the baseline period, while conditions related to pain, substance use disorder, insomnia, and cardiovascular disease were also prevalent.

**Table 1:** Patient cohort characteristics and comorbid conditions

Characteristic	Value
Age (years), Mean (SD)	45.4 (20.1)
18–19 years old	9.2%
20–24 years old	14.7%
25–34 years old	13.0%
35–44 years old	12.6%
45–54 years old	14.0%
55–64 years old	15.3%
65–74 years old	13.2%
75+ years old	8.1%
Gender: Female	57.2%
Post-index follow-up time, Mean (SD)	265.0 (127.0)
Proportion of patients with at least __ days of follow-up:	
≥ 30	95.0%
≥ 180	71.2%
≥ 365	51.5%
Charlson comorbidity index score, Mean (SD)	2.09 (3.14)
Comorbid conditions (1-year pre-index period)	
Anxiety disorder	40.7%
Essential hypertension	36.4%
Hyperlipidemia	24.2%
Chest pain	21.1%
Low back pain	20.9%
Substance use disorder (not incl. nicotine)	19.8%
Abdominal pain	18.3%
Dyspnea	17.9%
Headache	15.6%
Gastroesophageal reflux disease	15.4%
Insomnia	14.1%
Nicotine dependence	14.0%
Chronic pain	13.8%
Urinary tract infectious disease	12.9%
Neck pain	12.5%
Fatigue	12.5%
Vitamin D deficiency	12.0%
Type 2 diabetes mellitus without complication	12.0%
Obesity	11.1%
Anemia	10.1%

Prior to the index SI/SA event, treatment with an antidepressant and/or related medication classes was common (Table 2). More than 40% of individuals filled a prescription for an SSRI in the year prior, more than a third received an anxiolytic, and anticonvulsants and the group of other antidepressants were each received by more than one-quarter of patients during this time.

**Table 2:** Medications received during the one-year pre-index period (indexdate-365 days to indexdate-1 day)

Medication	%
Treatment classes of interest	
SSRI	43.1%
Anxiolytic	34.7%
Anticonvulsant	27.9%
Other antidepressants	25.6%
Hypnotics and Sedatives	16.9%
SNRI	16.5%
Psychostimulant	7.2%
Tricyclic	5.5%
Atypical antipsychotic	5.3%
Lithium	0.4%
MAOI	0.1%

*SSRI* selective serotonin reuptake inhibitor, *SNRI* serotonin and norepinephrine reuptake inhibitor, *MAOI* monoamine oxidase inhibitor.

Out of all patients identified, 84.4% (*n* = 35,624) had at least one class-based regimen of interest at any time during the one-year post-index period (including the index date). Receiving multiple class-based regimens due to switching and/or adding therapy classes was common in this population. Of those receiving any treatment, 70.2% went on to a subsequent class-based regimen, 46.3% received a third, and 28.1% received at least four class-based regimens within 1 year following the index date (Table 3). Receipt of more than one treatment class simultaneously was also prevalent, occurring within more than half of patients.

**Table 3:** Number of class-based regimens received and simultaneous receipt of multiple treatment classes during each treatment regimen

	% of treated
Class-based regimens received	
≥ 1	100.0%
≥ 2	70.2%
≥ 3	46.3%
≥ 4	28.1%
Simultaneous receipt of multiple treatment classes	
During any regimen	76.5%
1st regimen	59.8%
2nd regimen	57.5%
3rd regimen	59.9%
4th regimen	63.7%

Within treated patients, SSRIs were the most commonly received medication class at any time over the next year (61.9%), followed by the group of “Other antidepressants” that include drugs such as bupropion and trazodone (51.3%), anxiolytics (50.8%) and anticonvulsants (43.6%) (Table 4). Use of ECT or TMS procedures was rare, < 2% of patients received either treatment during the one-year follow-up. Within the first class-based regimen received following the index date, SSRIs were the most common treatment class observed (49.6%), and often occurred in combination with other treatments, with 69.3% of patients who received a first-line SSRI also receiving another medication class simultaneously. As treatment regimens progressed, use of an SSRI declined and use of anticonvulsants, hypnotics and sedatives, and psychostimulants became more prevalent.

**Table 4:** Proportion of patients receiving each treatment class within each regimen

Treatment	Any regimen	1st regimen	2nd regimen	3rd regimen	4th regimen
Pharmacotherapies					
SSRI	61.9%	49.6%	43.9%	38.9%	36.5%
Other antidepressants	51.3%	37.8%	34.3%	34.9%	36.7%
Anxiolytic	50.8%	36.1%	31.5%	33.9%	35.0%
Anticonvulsant	43.6%	29.3%	33.4%	36.7%	39.6%
SNRI	26.9%	18.8%	20.0%	21.1%	22.9%
Atypical antipsychotic	20.1%	13.8%	4.7%	3.2%	2.8%
Hypnotics and Sedatives	19.1%	9.6%	11.0%	12.6%	14.7%
Psychostimulant	9.3%	4.6%	6.9%	8.5%	9.8%
Tricyclic	7.1%	3.6%	4.2%	4.9%	6.0%
Lithium	2.6%	1.3%	1.6%	2.0%	2.4%
MAOI	0.1%	0.1%	0.1%	0.0%	0.1%
Procedures					
Electroconvulsive therapy	1.4%	1.0%	0.5%	0.5%	0.5%
Transcranial magnetic stimulation	0.4%	0.1%	0.1%	0.2%	0.3%

*SSRI* selective serotonin reuptake inhibitor, *SNRI* serotonin and norepinephrine reuptake inhibitor, *MAOI* monoamine oxidase inhibitor.

**Fig. 1:**
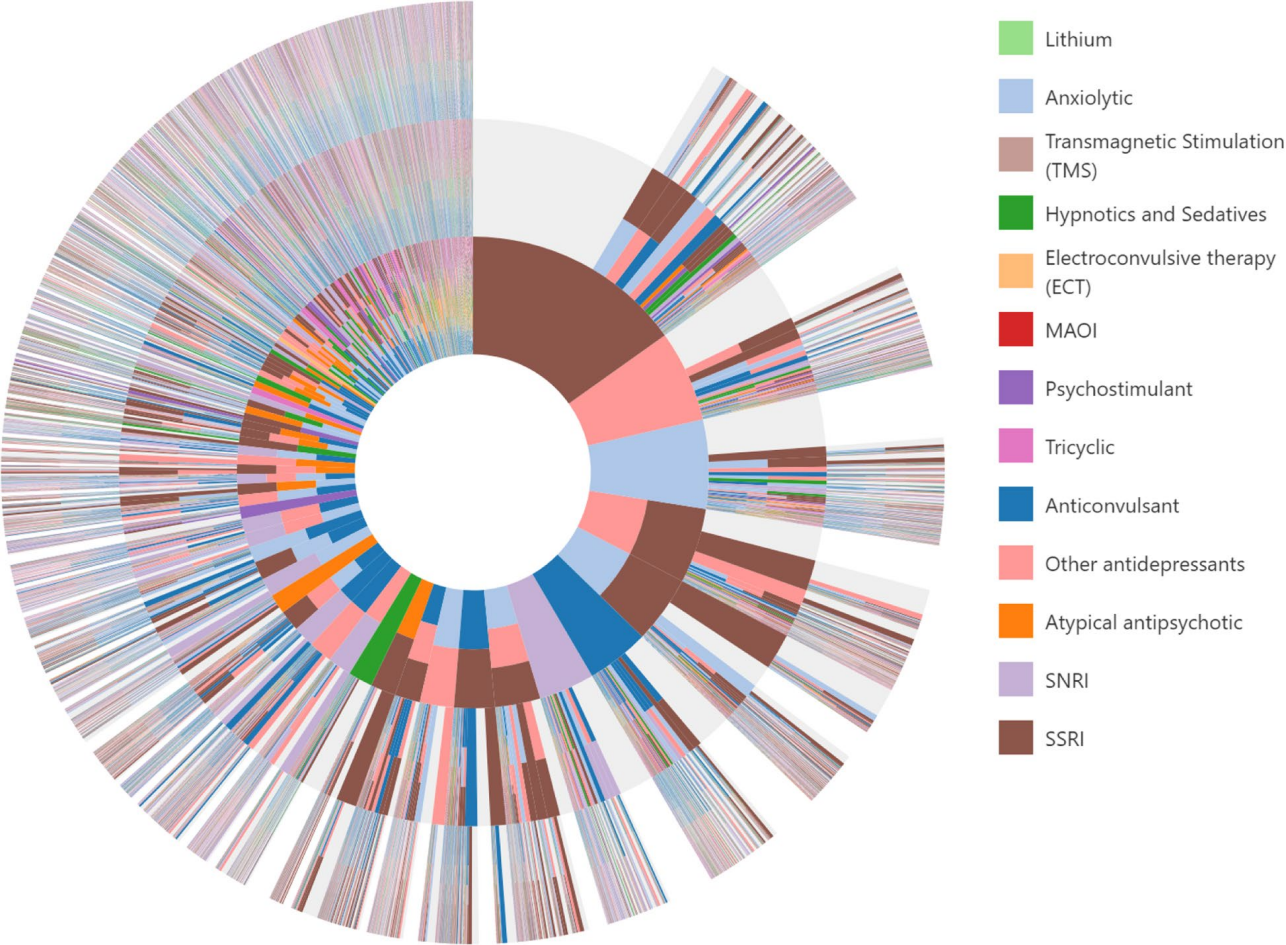
Sunburst of treatment patterns starting with first line (inner-most circle) to third line (outer circles). Each slice represents a treatment regimen, each color represents a distinct treatment class, and each layer represents a new regimen received and illustrates the sequence in which patients received different therapies; for example, the large brown piece in the top-middle indicates a first regimen of SSRI only, and the light blue slice on the next outer ring adjacent indicates a switch to an anxiolytic (2nd regimen). Slices that have multiple colors (e.g., brown + light blue) indicate a regimen of combination therapy with more than one medication class (e.g., SSRI + anxiolytic). Slices in light grey indicate no additional medication was taken. SSRI, selective serotonin reuptake inhibitor; SNRI, serotonin and norepinephrine reuptake inhibitor

The sequencing of the treatment classes was assessed and is displayed in Fig. 1. The inner circle of the sunburst plot illustrates the first class-based treatment regimens received after the index SI/SA event. Moving outward to the next ring represents the sequence from the first to second regimen. The most common first regimen received was SSRI therapy alone (15.2% of patients) as shown by the largest slice in the inner circle. From there, the majority of patients filled no other class-based regimens, while others continued use with an SSRI while adding on an anxiolytic as their second regimen, Other antidepressant, or anticonvulsant. Switching from SSRI therapy to an anxiolytic, Other antidepressant, or anticonvulsant is represented by the light blue, pink, and dark blue slices, respectively. The large number of slices and the high prevalence of multiple colors occurring within the same slice illustrate the variety of class-based regimens received by MDD patients following a suicide attempt or suicidal ideation.

## Discussion

There has been limited research examining the real-world pharmacologic treatment patterns and therapeutic procedures for MDD patients who have a suicide attempt or diagnosed suicidal ideation. Prior research examining the treatment of patients with suicidal ideation has been limited to cross-sectional associations with limited detail on the type of treatments that were received by patients [7, 30].

The current study utilized prescription and medical claims data from a commercially insured patient population representing a broad cross-section of the US population, including a portion of those receiving Medicare. The population had similar representation across all age groups indicating that suicidal ideation and suicide attempts are not limited to only younger adults.

There was a large amount of variability in treatment patterns. While SSRI use without concomitant use of any other treatment class was the most common first regimen received, it accounted for just 15% of regimens. The large majority of patients received combination therapy, and more than a quarter of the population were exposed to 4 different class-based regimens. The wide variety of classes used and the assorted combinations in which they were used likely reflect the characteristics of the patients, the array of comorbid conditions (e.g., anxiety and insomnia), the severity and refractoriness of MDD with SI/SA, and the challenges that health care providers face when taking care of patients who are exhibiting suicidal ideation or had a suicide attempt. Compared with prior research examining the general MDD population in the same database [21], this cohort of patients with SI/SA presents a much more complex treatment profile with higher rates of combination therapy, including regimens of three, four, or more simultaneous treatment classes, and more common use of non-antidepressant therapies such as anxiolytics and anticonvulsants. For example, in the newly diagnosed MDD population, just 18% of first-line treatment was combination therapy compared with more than triple that in the current study. Further, while more than one-quarter of patients with SI/SA received at least 4 distinct treatment regimens within just 1 year of follow-up, in the MDD population where patients were followed for a longer period of time (3 years), only 12% had at least 4 unique treatment regimens.

Anxiety and insomnia were commonly observed in these patients with MDD + SI/SA and we also observed how commonly anxiolytics and hypnotics/sedatives are prescribed. Alternatively, lithium is likely the most effective of these treatment options in preventing suicide [19, 31] but was received by less than 3% of patients in this study, while anticonvulsants were received by more than a quarter of patients following their SI/SA. This discrepancy between evidence and real-world practice is likely due to multiple factors – lithium may be thought of as an “older” treatment and therefore not as effective as newer treatment options, concerns about side-effects, overdose risk and other safety events, and that it could be considered off-label use [19].

A strength of this study was the inclusion of ECT and TMS procedures in addition to pharmacotherapy; typically claims-based research of treatment patterns focuses solely on prescription medications rather than procedures. While the prevalence of ECT and TMS were very low, knowing how often these options are utilized in the real-world is an important addition to the knowledge space.

This study includes data through March of 2020 and thus doesn’t include any treatments that came to market around that time or any time after. For example, in March 2019 esketamine was approved to treat treatment resistant depression [32] and in April 2020 it received approval for the treatment of depression in adults with MDD who have acute suicidal ideation or behavior [33]. Future research of treatment patterns, when sufficient data is available for esketamine and other newly approved treatments, may observe the impact of these therapies on treatment decisions. This study also did not capture the time between the start of one treatment to the start of the next; however, because maximum follow-up was just 1 year (mean < 9 months) and nearly half of patients received at least 3 regimens during this time, it can be assumed that average time on any single regimen was a few months.

Patients were identified using diagnoses codes which are not a perfect tool; however, we based our algorithm for identifying patients with suicide attempts on algorithms shown to have high validity (PPV ranging from 70 to 100%) [22, 23]. Similarly, a diagnosis for MDD was based on a definition shown to have high validity (PPV = 99%) [26]. Comorbid conditions, on the other hand, were identified via the requirement of a single diagnosis code, which allowed us to maximize the sensitivity for capturing these conditions. This analysis describes the order and frequency in which different pharmacotherapies are received at the class level, and as such it did not capture any within-class switching, frequency of switching back to a previously used medication class, or within-class combination use.

## Conclusions

This study fills a gap in knowledge by providing details on the real-world pharmacotherapy treatment practices following a suicide attempt or suicidal ideation; something that has been examined in very little detail until now. The high variability in treatment exposures, the large number of different treatment classes received by individual patients, and the high prevalence of concomitant use of multiple therapy classes reflect the complex nature of SI/SA and the comorbid profile of these individuals.

## Funding

This research did not receive any specific grant from funding agencies in the public, commercial, or not-for-profit sectors.

## Supplementary Information

**Additional file 1.**

**Additional file 2.**

## Data Availability

The data that support the findings of this study are available from Optum© but restrictions apply to the availability of these data, which were used under license for the current study, and so are not publicly available. Data are however available from the authors upon reasonable request and with permission of the data vendor.

## Abbreviations

ECT: Electroconvulsive therapy
ICD-9-CM: International Classification of Diseases, Clinical Modification codes, 9th revision
ICD-10-CM: International Classification of Diseases, Clinical Modification codes, 10th revision
IRB: Institutional Review Board
MAOI: Monoamine oxidase inhibitors
MDD: Major depressive disorder
OHDSI: Observational Health Data Sciences and Informatics
OMOP: Observational Medical Outcomes Partnership
PPV: Positive predictive value
SA: Suicide attempt
SI: Suicidal ideation
SNOMED-CT: Systematized Nomenclature of Medicine - Clinical Terms
SNRI: Serotonin and norepinephrine reuptake inhibitors
SSRI: Selective serotonin reuptake inhibitors
TMS: Transcranial magnetic stimulation
